# Off Service, with Purpose: Creating Learner-Centered Experiences for Off-Service Residents

**DOI:** 10.5811/westjem.65915

**Published:** 2026-11-02

**Authors:** Ivan Zvonar, Max Griffith, Alexander Garrett

**Affiliations:** * University of Washington School of Medicine, Department of Emergency Medicine, Seattle, Washington; † Wenatchee Emergency Physicians, Wenatchee, Washington

## Abstract

Academic emergency departments (EDs) host off-service residents (OSRs) from various specialties. While OSRs contribute meaningfully to patient care, they often face unique challenges during their ED rotations, including limited preparation, heightened stress, and unmet personal expectations. Despite a standardized curriculum endorsed by the American College of Emergency Physicians, guidance on how to foster meaningful educational experiences for OSRs remains sparse. This perspective offers a structured approach grounded in self-determination theory (SDT), which emphasizes autonomy, competence, and relatedness as essential to intrinsic motivation and effective learning. By aligning educational strategies with SDT principles, emergency medicine educators can enhance engagement, support learner development, and create lasting interprofessional collaboration. Ultimately, this learner-centered approach not only enriches the OSR experience but also strengthens the role of the ED as a dynamic, inclusive learning environment for all trainees.

## INTRODUCTION: OFF-SERVICE RESIDENTS IN THE EMERGENCY DEPARTMENT

Academic emergency departments (ED) are busy clinical areas owing to both high patient volumes and a vast spectrum of learners. In addition to emergency medicine (EM) residents, trainees from other specialties rotate through the ED either as part of their Accreditation Council for Graduate Medical Education (ACGME) program requirements 1–4 or for additional clinical experience. The American College of Emergency Physicians acknowledges the importance of ED training for off-service residents (OSR), and the Academic Affairs Committee has created a standardized OSR curriculum 5 —although this most recent national effort was more than 15 years ago. Little has been written about the OSR experience in the ED and how EM educators can foster a robust learning environment for them. Indeed, we owe all trainees an engaging experience, not only in recognition of their hard work that increases the productivity of EM faculty, 6 but also to serve as the foundation for their professional relationship with the ED in their future careers.

Off-service residents face numerous challenges regarding their EM rotations. They receive little to no didactic instruction tailored to their background, 7 are not adequately prepared to manage foundational EM patient complaints, 8 require more frequent faculty supervision, 9 and take significantly more time to reach the productivity level of an EM resident. 10,11 The ED can also be a stressful environment owing to high patient acuity, diagnostic uncertainty, and moral injury, 12 with OSRs more emotionally impacted than their EM counterparts. 13 Thus, it is unsurprising that OSRs frequently report that EM rotations fail to meet their expectations—a gap that may be the result of a one-size-fits-all model, as opposed to a more learner-centered approach. 14

A deliberate focus on motivation is a required component of a learner-centered clinical experience. 15 Self-determination theory (SDT) has emerged as the motivational theory best positioned to guide medical education advancement, because it reinforces autonomy and potentially leads to more effective healthcare delivery by trainees. 16 SDT specifically prioritizes learner-centeredness by addressing both intrinsic motivation and autonomous self-regulation—effectively getting at the *why* behind trainees’ actions—thereby providing a helpful framework for a more OSR-centered approach to EM rotations. 17 In this perspective, we review SDT’s three core psychological needs for learning—*autonomy, competence, and relatedness*—in the context of OSRs rotating in the ED. In doing so, we aim to improve the OSR experience by offering opportunities and best practices for supervisors with a basis in evidence and theory.

## SELF-DETERMINATION THEORY

Of several evidence-based models for motivation in education, 18 SDT has emerged as one of the most commonly used in health professions education research. 19 SDT posits that humans have an innate tendency toward psychological growth, development, and learning, and educational environments can either support or hinder these processes. 17 A central tenet of SDT is that intrinsic motivation and the internalization of autonomous self-regulation leads to better and more sustained learning. 20 This is in contrast to extrinsic motivation, which refers to behavior driven by external outcomes for which the learner does not hold a shared value. To support intrinsic motivation, a learning activity must fulfill the three fundamental needs of self-determination: autonomy, competence, and relatedness. 20 Autonomy is the feeling that the choice to do something arises from within the individual, rather than being told what to do. Competence refers to the desire to feel capable and effective at a given task. Relatedness is the need to feel a connection both to the work one is doing and to others in the learning environment.

It is the responsibility of educators in the ED to support the growth and development of all learners. By intentionally framing clinical tasks through the lens of autonomy, competence, and relatedness, we can empower OSRs while on rotation, and in doing so contribute to their growth as lifelong, self-regulated learners. Below, we offer suggestions and opportunities to support OSRs in the three key pillars of self-determination in the ED.

## LET THEM STEER: SUPPORTING AUTONOMY

While OSRs are training for careers distinct from EM, granting authentic learning opportunities in the ED through autonomy-supportive teaching promotes buy-in and maximizes engagement. This approach minimizes controlling teaching behaviors by focusing on understanding the learner’s viewpoint and providing them with choices and options, thereby allowing them to identify their own learning objectives. 21 Importantly, this does not equate with independence per se but rather emphasizes graduated responsibility. 22 In doing so, the learner acts in accordance with their personal values and interests in a supportive environment. 17 Operationalize this by beginning each shift discussing what the OSR’s key learning goals are for the day. This process of co-construction promotes self-regulation 23 and establishes an expectation that while they are providing a valuable service, they can also gain skills that will serve them in their future careers. Consider a way to track these goals, such as with shift cards, both to increase productivity 11 and provide an avenue for formative feedback.

Further support autonomy by allowing the OSR to step into the role of an emergency physician. Prompt them to commit to management plans despite their relative unfamiliarity with emergency care. Encourage them to take ownership over the care of their patients so that they feel and act as the primary clinician, 24 managing their patients to the extent that they are capable. Staff may look for a familiar face when they have questions or need orders, but they can be politely redirected to the OSR in these instances. This ownership should include the performance of procedures, when that is aligned with a learner’s goals. Because EM residents must meet key index procedure requirements set forth by the ACGME, 25 it can be tempting to preferentially offer them procedural opportunities. However, clinical educators are responsible for the development of all learners and should judiciously share opportunities with a consideration for equipoise and patient safety. Therefore, when an OSR’s patient requires a procedure, they should be given the opportunity to perform it so long as it is deemed safe. It is important for OSRs to gain experience with core procedures 5 because many procedural skills—such as those that use ultrasound guidance or Seldinger technique—translate to other specialties.

## CREATE A TOOLBOX: BUILDING COMPETENCE

Since OSRs come from various specialties and are often early in their training when they rotate in the ED, their foundational EM knowledge base is variable. Emergency medicine requires uniquely time sensitive evaluations of acute patient presentations. 5 Although medical competence is multilayered, OSRs’ goals during their ED rotations should target canonical EM knowledge and skills. 26 A targeted needs assessment can identify gaps in knowledge specific to OSRs at your institution. 8 This can guide the development of an OSR curriculum that best suits your department’s rotation. Alternatively, use a standardized curriculum that can be integrated into the rotation. 5 Didactics can be combined with residency conferences or added asynchronously. 27 Coordinate with EM chief residents and other residents in education roles to provide OSR orientations that cover essential EM topics and departmental operations, and address the practical needs that are essential in new healthcare settings (eg, where bathrooms and the cafeteria are located). 28 Consider providing introductory ultrasound teaching, because the application of point-of-care ultrasound is evolving and extending to specialties beyond EM. 29

Due to the expanding scope of knowledge required to practice EM, clinicians are increasingly using digital resources to answer clinical questions. 30 With the use of free open-access medical education, there is the danger of inappropriate representation of core content. 31 Make sure to provide access to foundational EM resources to establish a basis for self-directed learning. 32 Doing so will empower OSRs to look things up for themselves and thus prepare them for future learning. 33

Since EM encompasses a wide variety of patient presentations, OSRs will likely manage conditions for which they have preexisting knowledge due to their specific areas of training (eg, an ophthalmology resident seeing an eye complaint in the ED). When this occurs, encourage them to teach the team what they know about the topic. This will highlight the OSR as an asset to the team, build their context-dependent knowledge, and help develop personalized competence—higher order skills that lead to physician maturation. 26

## SEND AN INVITE: CULTIVATING RELATEDNESS

Each residency rotation presents new challenges, 34 and residents’ stress and well-being are impacted by team support, workplace demands, and learning opportunities. 35 The ED can negatively impact each of these domains owing to its inherent nature as a busy and sometimes chaotic environment, constructed of ad hoc teams. Furthermore, negative comparisons to EM peers, feelings of exclusion, and perceptions of task irrelevance (eg, *“Why do I need to know this?”*) can dismantle relatedness and motivation. Therefore, educators need to intentionally create an inclusive environment in which to frame OSRs’ connection to the tasks at hand.

To add further nuance to this context, OSRs are undergoing the process of forming their specialty identity, using self-categorization and social comparison to highlight similarities and differences between their specialty and others. 36 Furthermore, an absence of early interprofessional specialty collaboration can lead to future conflict. 37 Because EM is often described as a team sport, 38 OSRs must feel fully integrated into the clinical workflow. This lays the groundwork for future relationships when they return to the ED (eg, as a consultant or admitting clinician). Educators can promote this inclusion by introducing them to nurses, medical assistants, pharmacists, and other key professionals in the department. Because much of team building occurs outside of strict patient care, include OSRs in social conversations in the workplace and social gatherings outside the ED when appropriate.

It is also important to include OSRs in asynchronous learning opportunities and draw parallels to their future career contexts. Sharing interesting cases with OSRs, even when they are not caring for the patient, can further provide an opportunity to engage OSRs and have them reflect on shared interests in patient care. Although the alignment between a patient’s ED presentation and an OSR’s specialty may not be readily apparent, there are always learning points that can connect the dots. While we discourage the practice of “cherry picking” chief complaints that are familiar, 39 we do recommend highlighting how EM differs in its approach to patient presentations. For example, when working with a family medicine resident, one might discuss acute management of asymptomatic hypertension and how it differs from chronic outpatient hypertension management. Teaching can also highlight the ways in which an OSR’s specialty interfaces with the ED (eg, reviewing the appropriate organic medical workup for presumed decompensated psychiatric disease with a psychiatry resident). Additionally, it is important to highlight how the ED functions in supporting an OSR’s specialty—such as exploring how to use social resources to provide alternatives to admissions with an internal medicine resident—because their future jobs will interface with the ED.

## CONCLUSION: TOWARD A COLLABORATIVE FUTURE

Off-service residents are major contributors to academic emergency departments, and their time spent training in the ED is formative for both their medical knowledge and their understanding of the role of emergency medicine in healthcare. To create learner-centered experiences for OSRs in which they are intrinsically motivated, EM educators can provide opportunities for autonomous practice, assist in establishing competency in foundational EM topics, and create a welcoming environment that fosters both social and task relatedness, reinforcing the relevance of the ED rotation for their own specialty. It is important to note that the three core psychological needs of self-determination theory apply to all learners, including EM trainees. However, a tailored application of the techniques we have described can bolster the clinical learning of OSRs. These actions serve to both create productive rotations for OSRs and sustain healthy and collaborative relationships between EM and other specialties.
